# White Matter Microstructural Abnormalities in Children with Familial vs. Non-Familial Attention-Deficit/Hyperactivity Disorder (ADHD)

**DOI:** 10.3390/biomedicines13030676

**Published:** 2025-03-10

**Authors:** Rahman Baboli, Kai Wu, Jeffrey M. Halperin, Xiaobo Li

**Affiliations:** 1 Department of Biomedical Engineering, New Jersey Institute of Technology, Newark, NJ 07102, USA; 2 Graduate School of Biomedical Sciences, Rutgers University, Newark, NJ 07102, USA; 3 School of Biomedical Sciences and Engineering, South China University of Technology, Guangzhou International Campus, Guangzhou 511436, China; 4 Department of Psychology, Queens College, City University of New York, New York, NY 11367, USA; 5 Department of Electrical and Computer Engineering, New Jersey Institute of Technology, Newark, NJ 07102, USA

**Keywords:** familial ADHD, non-familial ADHD, white matter tract, diffusion tensor imaging, ABCD dataset

## Abstract

**Background**: Attention-deficit/hyperactivity disorder (ADHD) is a highly prevalent, heterogeneous neurodevelopmental disorder. **Methods**: This study presents, for the first time, a comprehensive investigation of white matter microstructural differences between familial ADHD (ADHD-F) and non-familial ADHD (ADHD-NF) using advanced diffusion tensor imaging analyses in a large community-based sample. **Results**: Children with ADHD-F exhibited significantly greater volume in the right anterior thalamic radiations and the left inferior fronto-occipital fasciculus compared to controls, and greater volume in the left inferior longitudinal fasciculus relative to ADHD-NF. The ADHD-NF group showed reduced fractional anisotropy in the left inferior longitudinal fasciculus compared to the controls. In both the ADHD-F and ADHD-NF groups, a greater volume of anterior thalamic radiation significantly contributed to reduced ADHD symptoms. **Conclusions**: Our findings suggest that white matter microstructural alterations along the frontal-thalamic pathways may play a critical role in hereditary factors among children with ADHD-F and significantly contribute to elevated inattentive and hyperactive/impulsive behaviors in the affected children.

## 1. Introduction

Attention-deficit/hyperactivity disorder (ADHD) is a neurodevelopmental disorder with a high prevalence, inheritability, and heterogeneity [1]. It is characterized by age-inappropriate inattention and/or hyperactivity-impulsivity for at least 6 months in multiple settings, such as home and school, with three different clinical presentations: predominantly inattentive, predominantly impulsive-hyperactive, or combined [2,3]. The onset of ADHD in children typically occurs before the age of 12, affecting nearly 11.4% of US children aged 3–17, and approximately 15% to 65% of individuals with a childhood diagnosis continue to be affected by this disorder into adulthood [4]. This persistence is reflected either through a continued ADHD diagnosis or by the presence of significant symptoms that do not fully meet the criteria for a clinical diagnosis [5]. ADHD is considered a major public health issue in the US, with an overall incremental cost of USD 143–266 billion. It is associated with multiple health-related behaviors, including addiction, sleep difficulties, anxiety-related issues, mood disorders, increased rates of divorce, higher rates of suicidal ideation and attempts, criminality, academic underachievement, employment challenges, physical injuries, and accidents [6,7,8,9,10,11,12,13,14,15]. Identifying etiological risk factors for ADHD, particularly during childhood, could facilitate timely and more effective prevention and interventions for affected children before they enter adolescence, potentially preventing the persistence of ADHD symptoms into adulthood.

Based on numerous investigations, it has been consistently observed that among the complicated and interactive biological and environmental components contributing to ADHD, familial history emerges as one of the most significant and influential risk factors for the emergence and persistence of ADHD [16,17,18]. One study demonstrated that children with a positive family history of ADHD have a significantly higher risk (approximately 5-fold) of developing the condition compared to those without a family history [19]. Another study estimated the heritability of ADHD to be approximately 70% to 90% [20]. Furthermore, studies have reported that relative to those without a family history of ADHD (ADHD-NF), a child with ADHD with a positive family history (ADHD-F) has a 4-fold higher risk for ADHD symptoms to persist into adulthood [21,22]. These findings suggest that ADHD-F may represent a biologically distinct and more homogeneous subgroup of ADHD [23]. Studying common and distinct neural substrates of ADHD-F vs. ADHD-NF could facilitate the development of more tailored early preventions and interventions that target the differential underlying neural anomalies characteristic of ADHD-F and ADHD-NF.

Over the last three decades, existing imaging and clinical/behavioral studies in ADHD without controlling for familial factors have shown widely inconsistent results [24]. Recent studies applying advanced machine learning (ML) and deep learning (DL) techniques have exhibited similar inconsistencies. For example, recent studies that applied advanced ML/DL techniques in the same imaging modality and measurement metrics, and reporting frontal lobe anomalies in ADHD, have shown incongruent results in terms of altered locations, tissue types, and anomaly directions of the metrics [25,26,27,28,29,30]. Similarly, although multiple studies have supported impairments in executive functions (EFs) in ADHD mediated by the prefrontal lobe and frontoparietal circuits [31,32,33], broader investigations have found that only about 50% of those with ADHD were impaired on the most sensitive EF measures (i.e., Stop Signal Reaction Time) [33,34,35]. Without considering the significant impact of the neurobiological heterogeneity of the disorder, decades of research so far have not established any specific neural substrates that are essential to move the field toward neurobiologically targeted diagnoses and interventions of ADHD.

Despite the inconsistencies in the findings of neuroimaging studies in children with ADHD, there remains a significant gap in the literature related to the influence of family history. Only a limited number of clinical and neuroimaging studies have investigated the effects of family history, particularly in siblings or twins, highlighting the need for further research in this area. Nevertheless, neuroimaging and neuropsychological studies controlling for etiological and biological heterogeneities have reported relatively more consistent results. Chen et al. showed that in a sample of 14 monozygotic (MZ) twin pairs, children with ADHD had smaller right striatum and thalamus volumes compared to their co-twins, while no significant change was observed in the cerebral cortex volume [36]. In another neuroanatomical twin study, Castellanos et al. found lower caudate volume in the affected children relative to their unaffected co-twins in nine discordant twin pairs [37]. Using functional magnetic resonance imaging (fMRI), Van’t Ent et al. found that during the Stroop and Flanker task, 27 MZ twin pairs that were highly concordant for attention problems had lower activation in the dorsolateral prefrontal, parietal, and temporal brain regions [38]. Godinez et al. showed lower activations in the frontoparietal network regions in children with ADHD relative to their co-twins [39]. A study investigating neuroanatomical anomalies in individuals with ADHD and their unaffected siblings, compared to groups of matched healthy control subjects, reported alterations in gray matter (GM) volume in the region of the right inferior frontal gyrus [40]. An fMRI study revealed lower functional connectivity within the fronto-striatal network during go/no-go tasks in cohorts with ADHD and their immediate family members in comparison to typically developed controls [41]. These findings suggest that GM neuroanatomical and functional underdevelopment associated with the frontal lobe and its related pathways can be a heritable pattern in individuals with a positive family history of ADHD. However, none of these neural anomalies were necessarily linked to ADHD symptoms in the study samples. More importantly, these small-sample studies, without an independent clinical control group for comparison, could not robustly identify familial risk-specific neurobiological mechanisms in ADHD.

On the other hand, childhood ADHD has recently been characterized by widespread white matter (WM) alterations in the developmental brain [42]. Multiple studies have reported that children with ADHD exhibited significantly abnormal fractional anisotropy (FA) and radial diffusivity in specific brain regions, including the fronto-striatal tracts, the superior longitudinal fasciculus, the cingulum bundle, the cerebral peduncle, the posterior limb of the internal capsule, etc. [43,44,45]. Additionally, multiple other diffusion tensor imaging (DTI) studies have reported reduced WM volume and FA in the fronto-parietal, fronto-limbic, corona-radiate, and temporo-occipital tracts in children with ADHD [46,47,48,49,50]. While these studies have indicated anomalies in the WM structure in children with ADHD, the findings are again largely inconsistent. Despite the technical variations in data acquisition/analyses and sample-related differences, the etiological heterogeneity of ADHD can play a critical role in WM neurobiological differences in ADHD samples. Unfortunately, the WM microstructural characteristics of children with familial vs. non-familial ADHD have not yet been sufficiently investigated [51].

To address these gaps in the field, the present study aimed to leverage a large sample of three independent groups of children (ADHD-F, ADHD-NF, and controls) from the baseline data of the ongoing Adolescent Brain Cognitive Development (ABCD) Study to investigate the WM microstructural substrates characterizing ADHD-F vs. ADHD-NF. Our recent structural MRI and fMRI studies in independent groups of AHDH-F, ADHD-NF, and control children from the ABCD Study cohort found that children with ADHD-F have some unique patterns of neural alterations, i.e., abnormal GM structural network properties associated with precuneus and paracentral gyrus [52], as well as reduced activation laterality of inferior frontal gyrus during working memory, thalamic, and frontal GM abnormalities [53,54], all associated with elevated ADHD symptoms in ADHD-F. On the basis of the findings from our team and other groups [24,46,52,54,55,56], we hypothesized that relative to matched controls and children with ADHD-NF, preadolescent children with ADHD-F would exhibit WM morphometrical and microstructural deficits in the frontal lobe and the major WM tracts connecting the frontal lobe and other brain regions, particularly the thalamus and thalamus-related pathways. These deficits may potentially affect the functional and behavioral integrity related to attention and play a significant role in the elevated inattentive and/or hyperactive/impulsive symptoms in the affected children.

## 2. Materials and Methods

### 2.1. Participants

For the current study, de-identified clinical and neuroimaging data were obtained from the ABCD Study baseline pool. This 10-year longitudinal investigation is currently the largest prospective study to comprehensively track cognitive brain development and child health in the US. The study initially enrolled 11,785 children (ages 9–10) from a major collaboration between 21 research sites across the country and is following them into early adulthood. The study’s recruitment and design process carefully considered age, sex, and socioeconomic variables to better mirror the US sociodemographic levels to ensure that the sample reflected the nation’s diversity, which significantly strengthens the generalizability of the findings [57]. The data are publicly acquirable and downloadable through the National Institute of Mental Health Data Archive [58]. All procedures were approved by the Institutional Review Board as well as the central IRB at the University of California, San Diego. All the parents provided written informed consent, and informed assent was obtained from the children.

The original exclusion criteria of the ABCD Study baseline enrollment included a current diagnosis of schizophrenia, autism spectrum disorder (moderate or severe), mental retardation/intellectual disability, or alcohol/substance use disorder [58]. Additional exclusion criteria of the present study included a history of traumatic brain injury based on the Modified Ohio State University TBI Screen-Short Version [59], a gestational age below 28 weeks, a birth weight under 1.2 kg, or the use of non-stimulant psychotropic medication within the past three months. Subjects involved in the present study met all inclusion criteria: proficiency in English for the participants and either English or Spanish for their parents, intelligence quotient (IQ) above 80 measured by the NIH Toolbox Picture Vocabulary Task T-score [60], and the provision of complete and high-quality MRI data needed. Approval for the study was not required in accordance with local/national legislation (https://www.ecfr.gov/current/title-28/chapter-I/part-46 (accessed on 6 March 2025)).

### 2.2. ADHD Assessments

The ADHD diagnoses and symptoms were assessed using parent or guardian responses to the recently validated Kiddie Schedule for Affective Disorder and Schizophrenia (KSADS-5) according to the DSM-5 criteria [58,61]. Children who were marked according to the screening questions for a high risk of having an ADHD diagnosis were further administered with the KSADS-5 ADHD supplementary scales for diagnoses. The diagnosis of ADHD was based on the threshold of endorsing at least six out of nine symptoms of inattention (ADHD inattentive presentation), at least six out of nine symptoms of hyperactivity/impulsivity (ADHD impulsive/hyperactivity presentation), or those who met the criteria in both domains (ADHD combined presentation).

After applying the general inclusion, exclusion, and ADHD symptomatology criteria, children with ADHD were further divided into two distinct groups: ADHD-F and ADHD-NF. The ADHD-NF group comprised 137 children who met at least one ADHD presentation criterion, had no biological parents with a current diagnosis or past diagnosis of ADHD, or significant inattentive and/or hyperactive-impulsive symptoms (as indicated by the T-score exceeding 65 on any of the three DSM-5 ADHD-oriented scales in the Adult Self-Report (ASR)) [62], and who provided high-quality imaging data for analyses. Conversely, the ADHD-F group included 114 children who exhibited at least one ADHD presentation, had at least one biological parent with a current or past ADHD diagnosis or a T-score above 65 on one of the three DSM-5 ADHD-oriented scales based on ASR, and provided high-quality imaging data for analyses. Children with ADHD with unknown family medical conditions or poor imaging quality identified from the preprocessing steps were removed from the study.

**Figure 1:**
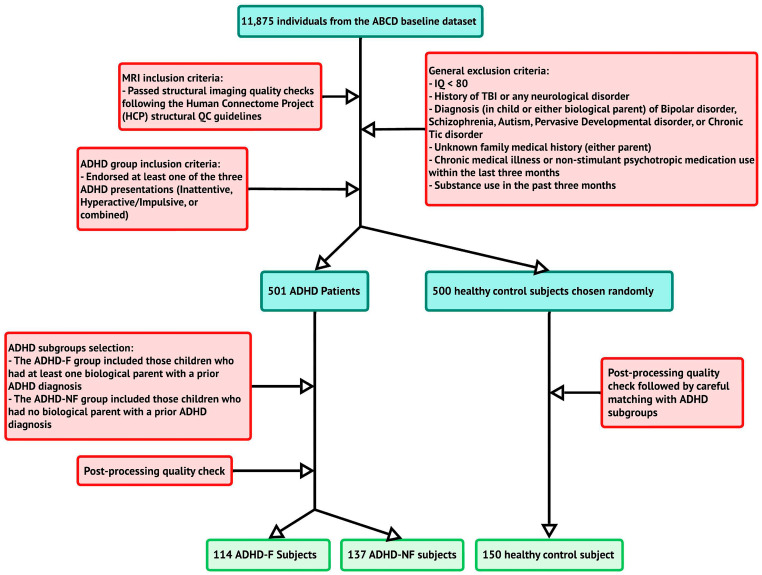
Flow diagram of the study cohort. ABCD: Adolescent Brain and Cognitive Development; ADHD-F: familial ADHD; ADHD-NF: non-familial ADHD.

Additionally, the third group, serving as the control group, included 150 children from the baseline pool of the ABCD study who did not present any symptoms of ADHD. This group was carefully matched for age, sex, handedness, IQ, and puberty category scale with the other two groups, ADHD-F and ADHD-NF. The detailed inclusion and exclusion steps are shown in Figure 1.

### 2.3. Demographic, Neurocognitive, and Clinical/Behavioral Measures

In this study, the Toolbox Picture Vocabulary Task (TPVT) was used as a measure of language ability, and an estimate of verbal intellectual was used as the IQ reference [60,63]. The Edinburgh Handedness Inventory was utilized to assess handedness [64,65]. The parent report of the Child Behavior Checklist (CBCL) was used for assessing behavioral and emotional problems [66]. Information about each participant’s medication was collected through the Parent Medication Survey Inventory, in which the parents were asked to provide the names and dosages of all medications their children had taken in the previous two weeks. All other demographic data, including age, sex, race, household income, and parental education, were collected from the demographic survey completed by each participant’s parent or guardian.

As we all know, the period from the pre-pubertal to post-pubertal stages is a critical time of human brain maturational changes, especially involving frontal GM and the WM tracts connecting cortical and subcortical structures [67,68,69,70]. These developmental changes in the human brain can introduce between-subject variability in studies of children with ADHD [68,69,71,72]. Therefore, this study proposed to use both age and the Puberty Category Score (PCS) to account for any imbalances in brain maturational stages across groups. The caregiver’s report of the ABCD Youth Pubertal Development Scale and Menstrual Cycle Survey History was used to assess the PCS [73,74].

### 2.4. Imaging Data Acquisition Protocol

The ABCD neuroimaging data were acquired at 21 different sites using 3T scanners (either Siemens, General Electric, or Philips, all with a 32-channel head). The neuroimaging data acquisitions and parameters have been previously described in detail [75]. The current study focused on diffusion-weighted imaging (DWI), but two other modalities of imaging, including T1- and T2-weighted structural MRI data, were used in the analyses as well. The following sequence parameters were used in the high angular resolution DWI: Repetition time/Echo time (TR/TE) of 4100/88 (msec) and 4100/81.9 (msec) for Siemens and General Electric, voxel size = 1.7 mm isotropic, 96 diffusion encoding directions, including 6 directions at b = 500 s/mm^2^, 15 directions at b = 1000 s/mm^2^, 15 directions at b = 2000 s/mm^2^, and 60 directions at b = 3000 s/mm^2^ repeated with inverted phase encoding (AP and PA), and field of view (FOV) = 256 × 256 mm^2^. T1-weighted structural MRI was acquired using an inversion prepared RF-spoiled gradient echo pulse sequence with the following parameters: Repetition time/Echo time (TR/TE) of 2500/2.88 (ms) and 6.31/2.9 (msec) for Siemens and General Electric, voxel size = 1.0 mm isotropic, field of view (FOV) = 256 × 256 mm^2^, matrix size = 256 × 256, flip angle = 8 (degree), and slice numbers = 176 and 208 for Siemens and General Electric scanners, respectively.

### 2.5. Individual-Level Imaging Data Preprocessing

The DWI from all participants was visually checked for any moderate to severe motion or any other artifacts, leading to the exclusion of those with such issues. Additionally, subjects who failed to score at least 3 on a 4-point scale (poor, fair, good, and excellent) for quality check, following the criteria of the Human Connectome Project Structural QC assessing image clarity, blurriness, and motion in T1-weighted structural images, were also removed from further analyses [76]. We used ABCD DTI-processed data. Diffusion MRI preprocessing steps include Eddy current correction [77], head motion correction [78], and spatial and intensity distortion correction [79]. Model-based Eddy current correction was used, focusing on displacements in the phase-encode direction. This approach models these displacements using 12 parameters, adjusting them based on gradient orientation and strength. To avoid errors caused by head motion resulting in dark slices and signal loss, a robust tensor fitting method was used to remove dark slices from the distortion estimation process. Those slices including signal loss (dark slices) were identified by calculating the root mean square (RMS) of residual errors. These values were then compared to the median value for every slice (frames with an RMS above a threshold were excluded from tensor fitting). This fitting process takes three iterations to stabilize, replacing the frame with signal loss (dark frames) with the synthesized images. Then, to optimize the Eddy current correction, Newton’s approach was used to minimize the RMS error between the corrected images and the synthesized images. And finally, following the correction, the tensor was re-estimated, removing the dark frames. This process was iterated five times for precision.

To correct for head motion, each frame was registered to a synthesized post-Eddy current correction volume. Slices with signal loss (dark frames) were excluded and replaced with interpolated values. And finally, the diffusion gradient matrix was adjusted for rotation.

To correct distortions from B0 field inhomogeneity, a robust technique involving phase-encoding polarity reversal is applied by aligning pairs of non-diffusion images with opposite encoding, a fast, nonlinear registration to adjust distortion across all frames [80]. The resulting displacement field is used to adjust each diffusion-weighted frame, followed by gradient nonlinearity correction. Then, the processed images are registered to T1-weighted structural images using mutual information after an initial alignment to a standard brain atlas and resampled to match the acquisition resolution, with adjustments for rotation and translation ensuring that all images fit a standard orientation across participants. Moreover, head rotation is accounted for during this process, and cubic interpolation is applied at each resampling step. And finally, a registration matrix records the rigid-body transformation between the dMRI and T1-weighted images, which allows for consistent diffusion orientations and improved data accuracy for subsequent analysis across the cohort.

### 2.6. Individual-Level Imaging Data Processing and Analyses

Briefly, AtlasTrack, a probabilistic atlas-based tool, was used to label major WM tracts, including additional pathways such as cortico-striate and cortico-cortical connections [78]. This atlas-based tool incorporates prior probabilities and orientation information for various long-range projection fibers. Structural MRI images for each subject are nonlinearly registered to the atlas using discrete cosine transforms, and the DTI orientations for each subject are compared to the atlas fiber orientations. This alignment process refines the a priori tract location probabilities and individualizes the fiber tract regions of interest (ROIs). Freesurfer’s “recon-all” function is used for automated brain segmentation, including skull stripping, volume registration, intensity normalization, WM segmentation, surface atlas registration, surface extraction, volumetric alignment to the MNI305 atlas, and final labeling of volume [81]. Then, multiple diffusion tensor measures are calculated, including FA and mean diffusivity (MD), using log-transformed diffusion weighted signals, providing important insights into the microstructural properties of the WM tracts [82]. Both the DTI inner shell model, which excludes data from frames with higher b-values (1000 s/mm^2^), and the DTI full shell, which includes a broader range of gradient strength, were fitted to ensure accurate assessments of the tissue’s microstructural properties across participants.

### 2.7. Secondary Tract-Based Spatial Statistics (TBSS) Analysis

**Figure 2:**
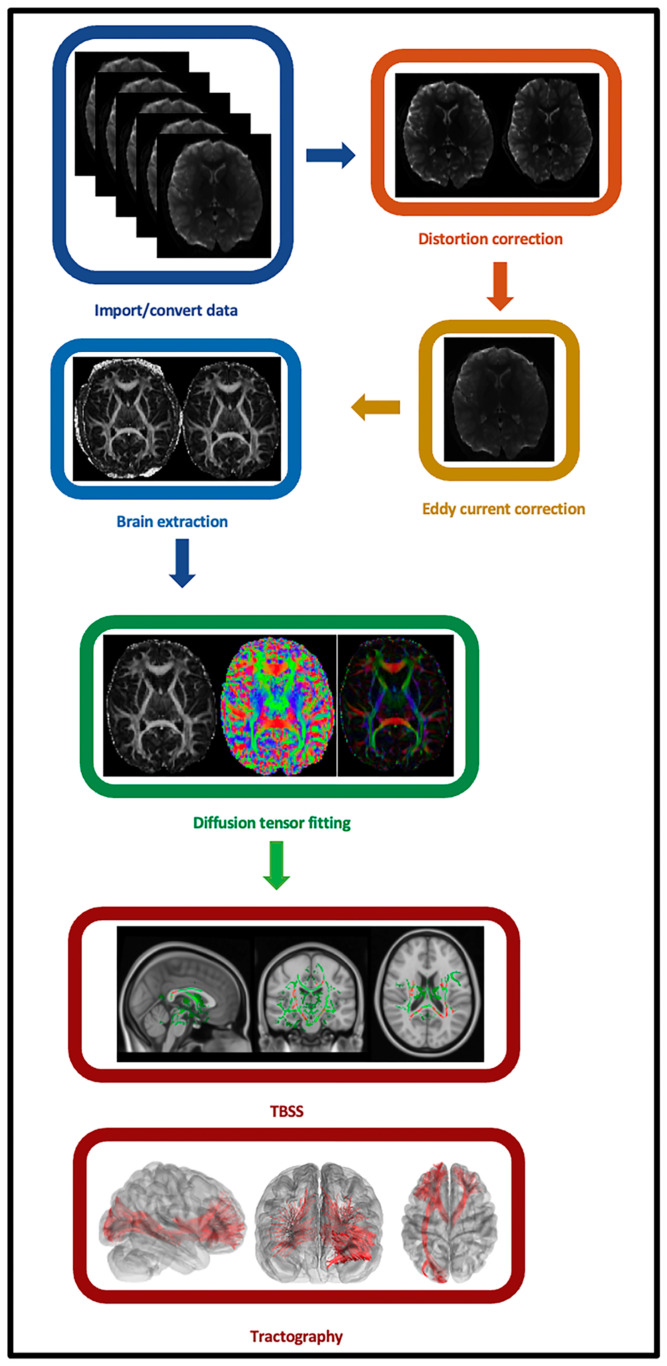
Overview of the study methods.

FA maps were processed using FSL (version 5) following a series of steps that have been described in detail in prior studies [83]. Briefly, the FMRIB58_FA template in the MNI common space served as the reference image for non-linear registration of all participants’ FA maps, employing the FNIRT tool (https://fsl.fmrib.ox.ac.uk/fsl/docs/#/structural/index (accessed on 6 March 2025)). Then, the transformed FA maps in MNI space were averaged and skeletonized to form a primary WM skeleton for all the participants. A threshold of FA ≥ 0.2 was applied to the averaged FA map to capture the inclusion of key WM tracts while eliminating non-WM ones. And finally, each participants’ aligned FA map was projected onto this skeleton, where each voxel was assigned the maximum FA value from a line perpendicular to the local skeleton. Figure 2 presents the graphical abstract and a summary of the study’s methods for individual-level analyses.

### 2.8. Group-Level Statistical Analyses

For the demographic, neurocognitive, and behavioral measures reported in Table 1, we performed one-way ANOVA or chi-square tests depending on whether the variable was continuous or categorical. For continuous variables meeting normality (Shapiro–Wilk test) and homogeneity of variances (Levene’s test), one-way ANOVA was used across the three groups (ADHD-F, ADHD-NF, and control). Categorical variables were analyzed using chi-square tests when Cochran’s rule was met, and Fisher’s exact test when it was not. For all RxC tables, pairwise comparisons were performed using Fisher’s exact test in addition to chi-square tests, as detailed in Supplementary Table S1. All analyses were conducted in SPSS (IBM Corp., Released 2020, Version 27.0, Armonk, NY, USA (with statistical significance set at *p* < 0.05)). In our primary ROI-based analyses, a mixed-model analysis of covariance (ANCOVA) was conducted to compare the FA and volume of each WM fiber tract among the three groups (ADHD-F, ADHD-NF, and control), with age, sex, handedness, IQ, and estimated intracranial volume (ETIV) as covariates. Bonferroni correction for multiple comparisons (at a corrected α = 0.05) was applied to control for potential false positives in these group comparisons. Brain–behavioral relationships in the ADHD probands were performed using Pearson correlation between the ROI-based WM measures that showed significant between-group differences and the ADHD symptom measures derived from CBCL and ksads-5 (*p*-values below 0.05 were considered statistically significant). These ADHD symptom measures included the t-score for ADHD-related problems on DSM-oriented CBCL scales and the raw scores for inattentive and hyperactivity-impulsivity symptom scores collected from KSADS-5. To compare the voxel-based maps of fractional anisotropy (FA) in white matter (among the three groups of ADHD-F, ADHD-NF, and control), we utilized Tract-Based Spatial Statistics (TBSS) analysis with a non-parametric, permutation-based threshold-free cluster enhancement (TFCE) approach in FSL. Specifically, the averaged FA skeleton (with a threshold at FA ≥ 0.2) served as the analysis mask, and we conducted 1000 permutations to build the null distribution. A significance threshold of *p* < 0.05 (family-wise error [FWE] corrected) was then applied, ensuring that the Type I error rate remained appropriately controlled for multiple comparisons. This TFCE-based FWE correction is a standard procedure in neuroimaging research and helps mitigate the risk of false positives across voxel-wise tests [78,79]. To assess the distributional properties of white matter tract measures, Hartigan’s dip test for unimodality was conducted on all significant white matter tracts across the ADHD-F, ADHD-NF, and control groups (using R package “dip.test”) [84].

## 3. Results

The demographic and clinical characteristics of the study participants are displayed in Table 1. The group comparisons did not reveal any significant between-group differences among the ADHD-F, ADHD-NF, and control groups in terms of age, sex, handedness, puberty category score, IQ, race, income, parental education, or ADHD presentations. Regarding medication status, the majority of participants in both the ADHD-F and ADHD-NF groups were either on no medication or on stimulant medication, with no significant differences in medication distribution between the two ADHD subgroups (*p* = 0.608). A smaller proportion of individuals in these groups were on non-stimulant medication or mixed medications, highlighting a comparable pattern of medication usage across the ADHD-F and ADHD-NF groups.

As displayed in Table 2, there were notable differences in WM tract fractional anisotropy values between the ADHD-F and control groups, particularly in the Forceps major (F(1,285) = 11.03, *p* = 0.023). Additionally, significant differences in fractional anisotropy were observed between the ADHD-NF and control groups in the left inferior longitudinal fasciculus (F(1,249) = 13.19, *p* = 0.001), indicating specific structural variations in this tract among the groups.

The WM tracts that exhibited significant between-group differences in volume are outlined in Table 3. The ADHD-F group demonstrated significant differences in the volume of the left inferior longitudinal fasciculus (F(1,249) = 11.34, *p* = 0.008) and right anterior thalamic radiation (F(1,262) = 13.80, *p* = 0.001) compared to the ADHD-NF and control groups, respectively. Furthermore, significant volumetric differences were detected in the left anterior thalamic radiation and left inferior-fronto-occipital fasciculus between the ADHD-F group and the control group (F(1,262) = 10.69, *p* = 0.007 and F(1,262) = 11.10, *p* = 0.005, respectively). These results highlight distinct structural differences in the WM tracts of children with ADHD-F relative to those with ADHD-NF and the control groups.

**Figure 3:**
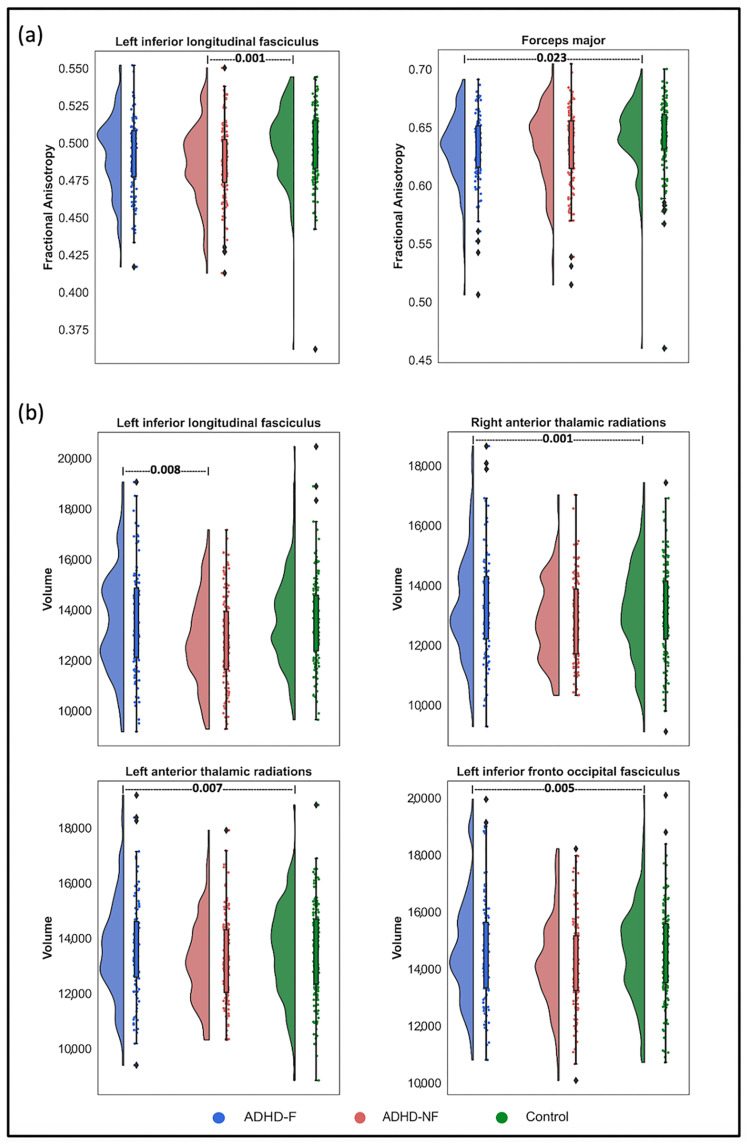
Raincloud plots of the (**a**) fractional anisotropy and (**b**) volume measures in white matter tracts showing significant group differences among the ADHD-F, ADHD-NF, and control groups. The half-violins illustrate each group’s data distribution, while the boxes depict the median and the 25th and 75th percentiles. Whiskers extend to 1.5 times the interquartile range beyond the box boundaries and cover 95% of observed data.

In order to visualize the distribution of FA and volume measures in regions that exhibited significant differences between groups (ADHD-F, ADHD-NF, and control), we used Raincloud plots. These plots show the distribution of these measures with jittering, median, interquartile range (IQR) boxes, and 95% confidence intervals (CI95). Figure 3 presents these Raincloud plots, which provide a clear depiction of how the ADHD-F, ADHD-NF, and control groups compare in terms of FA and volume measures across the significantly different regions. The plots also highlight the spread of the data and include the CI95 intervals, allowing for a better understanding of the variability and precision of these measures across the groups.

**Figure 4:**
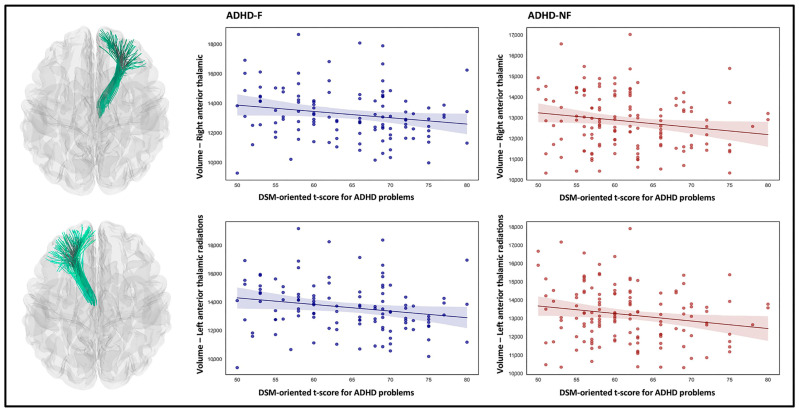
Regions that showed significant brain–behavior correlations in the ADHD-F and ADHD-NF groups. Each scatter plot depicts the relationship between the DSM-oriented ADHD T-score and the corresponding volumetric measure, along with a regression line and a 95% confidence interval (shaded band).

Brain–behavioral correlation analyses showed that in both groups of children with ADHD-F and ADHD-NF, higher volumes of the right anterior thalamic radiation (r = −0.193, *p* = 0.039 ADHD-F; r = −0.176, *p* = 0.039 for ADHD-NF) and left anterior thalamic radiation (r = −0.206, *p* = 0.028 for ADHD-F; r = −0.183, *p* = 0.033 for ADHD-NF) were significantly associated with lower T-score for ADHD-related problems on DSM-oriented CBCL scales (Figure 4).

The secondary whole-brain TBSS analysis revealed no significant differences between the three groups of ADHD-F, ADHD-NF, and controls.

Hartigan’s dip test for unimodality revealed no significant evidence of bimodality in any of the significant white matter regions (*p* > 0.05 for all tests). The full results of the dip test are presented in Supplementary Table S2.

## 4. Discussion

The present study aimed to explore the WM microstructural substrates linked to symptom manifestations in children with ADHD-F vs. ADHD-NF by leveraging a statistically robust sample of three independent groups—ADHD-F, ADHD-NF, and control—drawn from the baseline data pool of the ongoing ABCD Study. To the best of our knowledge, this study is the first in the field to assess the common and distinct WM microstructural substrates associated with symptom onset in ADHD-F vs. ADHD-NF.

Compared to matched controls, we found that the ADHD-F group had significantly increased WM volume of both the right and left anterior thalamic radiation tracts. While the ADHD-NF group showed an opposite trend of decreased WM volume anterior thalamic radiation compared to the ADHD-NF and control groups, the wide within-group variance demeaned the significance of the between-group differences. Furthermore, in both the ADHD-F and ADHD-NF groups, the higher WM volumes of both right and left anterior thalamic radiation tracts were significantly correlated with lower scores of ADHD problems based on the DSM-oriented T-score of the CBCL scale. The anterior thalamic radiation fiber bundle includes pathways that connect the mediodorsal thalamic nuclei and the frontal cortical regions, such as the prefrontal cortex, and is part of the fronto-striato-thalamic circuit, which plays a critical role in executive functioning, planning, complex behaviors, and social behaviors [85,86,87]. Substantial structural MRI studies have suggested that children with ADHD exhibit inconsistent anomalies in the anterior thalamic radiation, including alterations in the volume and fractional anisotropy (FA) of this WM tract [55,88,89,90,91]. A number of clinical and multi-modal neuroimaging studies in children with ADHD have demonstrated that structural and functional anomalies associated with the thalamus and frontal lobe can significantly contribute to the emergence of ADHD symptoms [40,55,92,93,94]. Family-based studies of ADHD have reported more convergent findings in terms of the functional and anatomical alternations associated with the frontal-thalamo pathways. For instance, neuroanatomical studies in siblings have reported that, compared to controls, both patients with ADHD and their unaffected siblings exhibit smaller frontal GM volumes [40,95]. Additionally, studies investigating functional and structural pathways have found reduced activation in the frontal regions and associated networks, which correlate with lower WM integrity in the prefrontal cortex [41,96,97]. Taken together with these existing findings, our results suggest that WM disruptions in the frontal-thalamo pathways, along with the GM functional and structural alterations, have a strong heredity role in children with ADHD-F and are significantly linked to symptom onset.

Our results also demonstrated that compared to matched controls, children with ADHD-F exhibited significantly reduced WM volume of the left inferior-fronto-occipital fasciculus. The inferior fronto-occipital fasciculus is a critical structural component of spatial attention. Anatomically, it originates from the posterior area of the occipital lobe, extends through the temporal lobe, and terminates in orbitofrontal and prefrontal cortices, connecting substantial brain regions of the ventral attention network [98,99,100]. Numerous clinical and neuroimaging studies have suggested that abnormalities in the WM tract contribute to behavioral symptoms of inattention in both children and adults with ADHD. For instance, a recent DTI study by Zhou et al. reported significantly reduced FA in children with ADHD compared to healthy control individuals [101], while Tremblay et al. observed a trend of increased FA in children with ADHD compared to controls [102]. Another family-based multi-modal neuroimaging study in ADHD reported significant differences in FA associated with the inferior fronto-occipital and uncinate fasciculi, suggesting a phenotypical correlation and shared genetic determination of the inferior fronto-occipital fasciculus WM tract [103]. Conversely, another imaging study using refined DTI tractography and investigating WM microstructure in subjects with ADHD and their unaffected siblings found no significant difference in DTI microstructural measurements of the inferior fronto-occipital fasciculus WM tract in children with ADHD, unaffected siblings, and controls [51]. Building on prior research, which has yielded inconsistent findings, our study, conducted in three large and independent groups of subjects, suggests that WM microstructural alterations in the left inferior fronto-occipital fasciculus may have a strong hereditary origin that characterizes a biologically unique long-distance WM connection pattern in children with ADHD-F.

Our results also revealed that the ADHD-NF group exhibited significantly reduced FA in the left inferior longitudinal fasciculus compared to the matched control group. This ventral associative WM tract, which connects the occipital and temporal lobes, plays a crucial role in the ventral semantic system, supporting functions such as visual processing, memory, and language. The observed FA reduction in this tract aligns with findings from previous studies. For instance, Gonzalez-Madruga et al. reported lower FA in the left inferior longitudinal fasciculus among patients with ADHD compared to healthy controls [104], while Svatkova et al. identified higher FA in the inferior longitudinal fasciculus in ADHD cohorts compared to healthy controls [90]. Again, discrepancies in the findings associated with WM abnormality of the inferior longitudinal fasciculus in ADHD can be sourced from technical variations in data acquisition and analyses, differences in sample characteristics, as well as heterogeneity in ADHD. Derived from comparisons in independent groups of ADHD-F, ADHD-NF, and controls, our findings suggest that underdeveloped WM structure of the left inferior longitudinal fasciculus may exhibit a more common neuroanatomical profile in children with ADHD who do not carry the family/genetic risk factor. While this neurobiological alteration in ADHD-NF may be linked to other cognitive or behavioral impairments, it does not necessarily play a role in inattention and/or hyperactivity-impulsivity in the affected children that characterize the disorder of ADHD.

## 5. Limitations and Future Directions

The results of this study should be considered while taking potential limitations into account. One limitation of this study is that only one parent was interviewed and participated in the ADHD-related assessments in the ABCD Study. Detailed medical information on other first-degree family relatives was acquired based on the responses of the interviewed parents to the family history questionnaire. To reduce the potential impact of inaccurate biological parents’ medical history information on our grouping of ADHD-F and ADHD-NF children, we excluded nearly half of the ADHD children originally identified from the ABCD Study baseline pool. Second, the ADHD assessments, based on parent-reported KSADS and supplementary scales, were intended for research purposes and were not clinically verified. Consequently, the findings should be further validated in a clinical sample. In addition, children with ADHD have diverse developmental trajectories and outcomes due to uneven brain development statuses and complex environmental interactions. Therefore, future longitudinal studies are expected to test the role of these early childhood neural markers of ADHD-F vs. ADHD-NF in different developmental stages, such as late adolescence and early adulthood, in affected individuals.

## 6. Conclusions

In conclusion, together with existing findings, the results of this novel research indicate that WM microstructure in tracts that connect regions of the prefrontal cortex and thalamus may have a strong heredity component in ADHD-F and play a critical role in symptom manifestations in affected children. Meanwhile, disrupted WM microstructures of the left inferior longitudinal fasciculus may represent a common neural deficit in ADHD-NF, which is significantly vulnerable to the environmental risk factors of ADHD. These findings are derived from large independent groups of subjects, providing more than sufficient statistical power for our findings. Future longitudinal research could build upon these insights to further understand the developmental trajectories of these specific neural substrates linked to ADHD-F vs. ADHD-NF.

## Figures and Tables

**Table 1:** Demographic and clinical characteristics of the study cohort (continuous variables as mean [±95% CI]; categorical variables as N (%) [CI range]).

	ADHD-F Mean (±95% CI) or N	ADHD-NF Mean (±95% CI) or N (%) [CI Range]	Control Mean (±95% CI) or N (%) [CI Range]	F or χ^2^	*p*-Value
Age (months)	119.90 [118.51, 121.29]	118.07 [116.77, 119.37]	119.16 [117.97, 120.35]	1.91	0.148
Sex:				2.54	0.280
Female	38 (34) [24.6, 42.0]	59 (43) [36.8, 49.4]	60 (40) [32.2, 47.8]		
Male	76 (66) [58.0, 75.4]	78 (57) [50.6, 63.2]	90 (60) [52.2, 67.8]		
Handedness:				2.40	0.662
Right-Handed	86 (76) [ 67.5, 83.37]	110 (80) [73.6, 86.9]	124 (83) [76.7, 88.7]		
Left-Handed	7 (6) [1.7, 10.6]	8 (6) [1.9, 9.8]	8 (5) [1.7, 9.3]		
Both-Handed	21 (18) [11.3, 25.6]	19 (14) [8.1, 19.6]	18 (12) [6.8, 17.2]		
Puberty category score:				-	0.548
Pre-Pubertal	65 (57) [47.9, 66.1]	80 (58) [50.1, 66.7]	91 (61) [52.8, 68.5]		
Early-Pubertal	30 (26) [18.2, 34.4]	26 (19) [12.4, 25.6]	36 (24) [17.2, 30.8]		
Mid-Pubertal	18 (16) [9.0, 22.5]	30 (22) [14.9, 28.8]	21 (14) [8.5, 19.5]		
Late-Pubertal	1 (1) [0.0, 2.6]	1 (1) [0.0, 2.1]	2 (1) [0.0, 3.2]		
IQ (Picture Vocabulary)	109.85 [106.29, 113.42]	105.39 [102.41, 108.37]	107.43 [104.87, 109.99]	2.02	0.133
Race:				1.72	0.943
Caucasian	85 (75) [67.0, 82.2]	101 (74) [66.1, 81.3]	112 (75) [67.5, 81.9]		
African-American	11 (10) [4.3, 14.9]	18 (13) [7.4, 18.8]	14 (9) [4.6, 14.0]		
More than one race	12 (10) [5.0, 16.1]	12 (9) [4.0, 13.6]	15 (10) [5.2, 14.8]		
Other races	6 (5) [1.2, 9.4]	6 (4) [0.9, 7.9]	9 (6) [2.2, 9.8]		
Annual income:				6.50	0.164
<USD 50,000	51 (45) [35.7, 53.6]	44 (32) [24.3, 39.9]	60 (40) [32.2, 47.8]		
USD 50,000–10,0000	24 (21) [3.7, 28.5]	28 (20) [16.5, 32.3]	24 [(16) [10.2, 21.8]		
>USD 100,000	39 (34) [25.3, 43.1]	65 (48) [39.3, 55.5]	66 (44) [36.4, 51.6]		
Parental education:				6.12	0.633
No high school diploma	4 (4) [0.1, 6.9]	8 (6) [1.8, 9.8]	11 (7) [3.0, 11.5]		
High school diploma	6 (5) [1.2, 9.4]	9 (7) [2.4, 10.8]	16 (11) [5.7, 15.7]		
Some college	40 (35) [26.3, 43.8]	45 (33) [24.7, 40.9]	47 (31) [23.8, 38.8]		
Bachelor’s degree	31 (27) [19.0, 35.5]	42 (31) [22.7, 38.6]	42 (28) [20.5, 35.7]		
Graduate degree	33 (29) [20.6, 37.3]	33 (24) [16.9, 31.4]	34 (23) [15.7, 29.7]		
Medication status:				-	0.619
No medication	81 (71) [62.7, 79.3]	104 (76) [68.5, 83.3]	-		
Stimulant medication	31 (27) [18.6, 35.8]	29 (21) [14.0, 28.4]	-		
No stimulant medication	1 (1) [0.0, 2.6]	3 (2) [0.0, 4.7]	-		
Mixed medications	1 (1) [0.0, 2.6]	1 (1) [0.0, 2.1]	-		
ADHD symptom presentation:				1.97	0.373
Inattentive	48 (42) [32.9, 51.3]	48 (35) [27.0, 43.0]	-		
Hyperactive-Impulsive	12 (11) [4.8, 16.1]	21 (15) [9.1, 21.6]	-		
Combined	54 (47) [37.9, 56.9]	68 (50) [41.3, 58.0]	-		

ADHD-F: familial ADHD; ADHD-NF: non-familial ADHD; IQ: intelligence quotient; CI: confidence Interval. Continuous variables (age, IQ) were compared by one-way ANOVA; categorical variables were compared by chi-square if Cochran’s rule was met, or Fisher’s exact (Freeman–Halton) if not.

**Table 2:** White matter tracts that showed significant between-group differences in fractional anisotropy with age, sex, handedness, IQ, and ETIV controlled for confounding factors.

White Matter Tract	Fractional Anisotropy			*p*-Value After Bonferroni Correction		
	ADHD-F (±95% CI)	ADHD-NF (±95% CI)	Control (±95% CI)	ADHD-F vs. ADHD-NF	ADHD-F vs. Control	ADHD-NF vs. Control
Left inferior longitudinal fasciculus	0.492 (0.487, 0.496)	0.486 (0.482, 0.491)	0.499 (0.495, 0.503)	0.145	0.076	0.001
Forceps major	0.631 (0.626, 0.637)	0.633 (0.628, 0.639)	0.642 (0.638, 0.647)	0.376	0.023	0.057

ADHD-F: familial ADHD; ADHD-NF: non-familial ADHD; IQ: intelligence quotient; ETIV: estimated intracranial volume; CI: confidence interval.

**Table 3:** White matter tracts that showed significant between-group differences in volume with age, sex, handedness, IQ, and ETIV controlled for confounding factors.

White Matter Tract	Volume			*p*-Value After Bonferroni Correction		
	ADHD-F (±95% CI)	ADHD-NF (±95% CI)	Control (±95% CI)	ADHD-F vs. ADHD-NF	ADHD-F vs. Control	ADHD-NF vs. Control
Left inferior longitudinal fasciculus	13,529 (13,157, 13,902)	12,796 (12,506, 13,086)	13,571 (13,288, 13,854)	0.008	0.150	0.064
Right anterior thalamic radiations	13,273 (12,950, 13,596)	12,843 (12,607, 13,061)	13,123 (12,872, 13,374)	0.188	0.001	0.193
Left anterior thalamic radiations	13,639 (13,309, 13,969)	13,202 (12,945, 13,459)	13,553 (13,287, 13,818)	0.209	0.007	0.283
Left inferior fronto-occipital fasciculus	14,643 (14,309, 14,977)	14,202 (13,942, 14,463)	14,599 (14,338, 14,860)	0.305	0.005	0.567

ADHD-F: familial ADHD; ADHD-NF: non-familial ADHD; IQ: intelligence quotient; ETIV: estimated intracranial volume; CI: confidence interval.

## Data Availability

All relevant data for this work are available from the corresponding authors upon reasonable request.

## Supplementary Materials

The following supporting information can be downloaded at: https://www.mdpi.com/article/10.3390/biomedicines13030676/s1, Table S1: Pairwise 95% confidence intervals for proportions and Fischer exact test results for categorical variables in Demographic and clinical characteristics of the study cohort. Table S2: Hartigan’s Dip Test results for unimodality assessment across significant white matter tracts. The test was conducted for Fractional Anisotropy and Volume measures in ADHD-F, ADHD-NF, and Control groups. The table presents the dip statistic (*D*) and associated *p*-value for each group.

## Abbreviations

ADHD	Attention-deficit/hyperactivity disorder
ADHD-F	ADHD with positive family history
ADHD-NF	ADHD without a family history of ADHD
ML	Machine learning
DL	Deep learning
EFs	Executive functions
MZ	Monozygotic
GM	Gray matter
WM	White matter
FA	Fractional anisotropy
DTI	Diffusion tensor imaging
ABCD	Adolescent Brain Cognitive Development
IQ	Intelligence quotient
KSADS-5	Kiddie Schedule for Affective Disorder and Schizophrenia
ASR	Adult Self-Report
TVPT	Toolbox Picture Vocabulary Task
PCS	Puberty Category Score
CBCL	Child Behavior Checklist
DWI	Diffusion-weighted imaging
TR	Repetition time
TE	Echo time
FOV	Field of view
RMS	root mean squared
MD	Mean diffusivity
TBSS	Tract-based spatial statistics
TFCE	Threshold-free cluster enhancement
