# Supplementary material for: White Matter Microstructural Abnormalities in Children with Familial vs. Non-Familial Attention-Deficit/Hyperactivity Disorder (ADHD)
Source: Biomedicines. 2025 Mar 10;13(3):676. doi: 10.3390/biomedicines13030676 (PMC11940736; doi:10.3390/biomedicines13030676)
Supplement: Supplementary file 1 [file biomedicines-13-00676-s001.zip › biomedicines-3434972-supplementary.pdf]

Table S1. Pairwise 95% confidence intervals for proportions and Fischer exact test results for categorical variables in Demographic and clinical characteristics of the study cohort.

	CI95 and p-value for Fisher's exact test ADHD-F Vs. ADHD-NF	CI95 and p-value for Fisher's exact test ADHD-F Vs. Control	CI95 and p-value for Fisher's exact test ADHD-NF Vs. Control
**Sex:**<br>Female<br>Male	(0.29, 0.42), 0.12	(0.29, 0.42), 0.30	(0.30, 0.43), 0.63
**Handedness:**<br>Right-Handed<br>Left-Handed<br>Both-Handed	(0.24, 0.49), 0.61	(0.24, 0.49), 0.80	(0.24, 0.49), 0.29
**Puberty category score:**<br>Pre-Pubertal<br>Early-Pubertal<br>Mid-Pubertal<br>Late-Pubertal	(-0.20, 0.87), 0.39	(-0.20, 0.87), 0.92	(-0.20, 0.87), 0.29
**Race:**<br>Caucasian<br>African-American<br>More than one race<br>Other races	(0.08, 0.44), 0.81	(0.08, 0.44), 0.9	(0.08, 0.44), 0.71
**Annual income:**<br><USD 50,000<br>USD 50,000-USD 10,0000<br>>USD 100,000	(0.17, 0.29), 0.07	(0.17, 0.29), 0.24	(0.31, 0.46), 0.33
**Parental education:**<br>No high school diploma<br>High school diploma<br>Some college<br>Bachelor's degree<br>Graduate degree	(0.18, 0.34), 0.78	(0.19, 0.35), 0.27	(0.14, 0.27), 0.76
**Medication status:**<br>No-Medication<br>Stimulant medication<br>No-Stimulant medication<br>Mixed-Medications	(-0.19, 1.19), 0.61	-	-
**ADHD symptom presentation:**<br>Inattentive<br>Hyperactive-Impulsive<br>Combined	(0.22, 0.35), 0.39	-	-

ADHD-F: familial ADHD; ADHD-NF: non-familial ADHD; IQ: Intelligence quotient; CI: Confidence Interval.

Table S2. Hartigan's Dip Test results for unimodality assessment across significant white matter tracts. The test was conducted for Fractional Anisotropy and Volume measures in ADHD-F, ADHD-NF, and Control groups. The table presents the dip statistic (*D*) and associated *p*-value for each group.

White matter tract	Measure	ADHD_F (D,p-value)	ADHD_NF (D,p-value)	Control (D,p-value)
Left inferior longitudinal fasciculus	Fractional anisotropy	**(0.026, 0.87)**	**(0.027, 0.70)**	**(0.026, 0.75)**
Forceps major	Fractional anisotropy	**(0.020, 0.99)**	**(0.024, 0.85)**	**(0.030, 0.45)**
Left inferior longitudinal fasciculus	Volume	**(0.024, 0.93)**	**(0.020, 0.97)**	**(0.024, 0.82)**
Right anterior thalamic radiations	Volume	**(0.024, 0.94)**	**(0.029, 0.57)**	**(0.020, 0.98)**
Left anterior thalamic radiations	Volume	**(0.020, 0.99)**	**(0.024, 0.88)**	**(0.019, 0.99)**
Left inferior fronto-occipital fasciculus	Volume	**(0.024, 0.93)**	**(0.020, 0.98)**	**(0.025, 0.76)**
